# Hemocompatibility Evaluation of PEGylated Bovine Hemoglobin

**DOI:** 10.3390/ijms27031262

**Published:** 2026-01-27

**Authors:** Linli Wang, Lian Zhao, Guoxing You, Weidan Li, Qiang Zeng, Hang Yu, Shiyu Tao, Hong Zhou, Yuzhi Chen, Ying Wang

**Affiliations:** Academy of Military Medical Sciences, Academy of Military Science of the Chinese People’s Liberation Army, Taiping Road 27, Haidian District, Beijing 100850, China; wanglinli19880822@163.com (L.W.);

**Keywords:** PEG-bHb, hemocompatibility, erythrocyte morphology, oxygen supply efficiency, coagulation function, immunocompatibility

## Abstract

Hemocompatibility is critical for intravenous hemoglobin-based oxygen carriers (HBOCs). We evaluated the hemocompatibility of polyethylene glycol-conjugated bovine hemoglobin (PEG-bHb) to facilitate its optimization and clinical translation. PEG-bHb was synthesized and characterized. In vitro hemocompatibility was assessed by incubating blood components with PEG-bHb (2.5–40 mg/mL), evaluating erythrocyte morphology/function, coagulation, complement activation, and leu kocyte phagocytosis. In vivo assessments in Wistar rats injecting PEG-bHb (50–100 mg/kg) included erythrocyte function, coagulation, complement activation, and histopathology. PEG-bHb exhibited increased hydrodynamic diameter, unaltered zeta potential, elevated colloidal osmotic pressure (COP) and viscosity, alongside a decreased P_50_ versus bovine hemoglobin (bHb). In vitro, PEG-bHb preserved erythrocyte morphology without inducing eryptosis, and oxygen supply efficiency was unaffected. Moreover, it slightly disrupted the activated partial thromboplastin time (APTT), the thrombin time (TT), and the platelet adhesion, while platelet activation and thromboelastography (TEG) remained unchanged. PEG-bHb did not activate complement and only mildly enhanced phagocytosis at 2.5 mg/mL. In vivo, PEG-bHb did not affect eryptosis, oxygen supply efficiency, coagulation, complement activation, and no inflammatory infiltration was observed. PEG-bHb maintains erythrocyte morphology and function, slightly perturb coagulation without compromising immunocompatibility, and demonstrated excellent hemocompatibility.

## 1. Introduction

Hemoglobin-based oxygen carriers (HBOCs) are used as blood substitutes, primarily in scenarios involving blood shortages or when blood transfusion is not feasible due to religious reasons [1]. However, early first-generation HBOCs, such as HemAssist, PolyHeme, and Hemolink, mainly prepared using polymerization or cross-linking techniques, failed to achieve successful clinical translation due to side effects, including hypertension, myocardial infarction, and renal impairment [2]. Hemopure, a glutaraldehyde-polymerized bovine hemoglobin, is a representative first-generation HBOC approved for acute blood loss in South Africa (2001) and anemia in Russia (2012); however, it still causes unavoidable vasoactive side effects [3]. Polymer-conjugated HBOCs are considered promising second-generation HBOCs. Among them, polyethylene glycol (PEG) has become a popular modifier due to its excellent biocompatibility, high water solubility, and strong hydration capacity [4]. The PEGylated hemoglobin shows prolonged plasma half-life, elevated colloid osmotic pressure (COP), low immunogenicity, and attenuated vasoactivity, primarily through an increase in its molecular volume and the masking of surface antigens [5]. Some PEGylated hemoglobin products have reached clinical trials. Hemospan (PEG-modified human hemoglobin) has been demonstrated to effectively carry oxygen in Phase I and Phase II clinical trials conducted in the United States [6,7]. Sanguinate (PEG-modified bovine carbonyl hemoglobin) has shown promise in early clinical trials for the treatment of sickle cell disease and has achieved the status of “Orphan Drug” under the FDA [8,9]. These advancements have established PEGylated hemoglobin as one of the most promising HBOCs.

To achieve improved treatment outcomes, we have developed a novel PEGylated bovine hemoglobin (PEG-bHb) in which each hemoglobin molecule is conjugated with an average of six 5 kD PEG chains. Since HBOCs are administered intravenously, they must demonstrate excellent hemocompatibility to support clinical applications such as the management of trauma and hemorrhagic shock. Consequently, a rigorous risk assessment in accordance with ISO 10993-4:2017/Amd 1:2025 is mandatory before clinical use, which includes analyses of hemolysis, cell counts, platelet and leukocyte activation, coagulation, and the complement system [10]. Previous studies have evaluated the hemocompatibility of several HBOCs. For instance, Kloypan C et al. demonstrated the excellent hemocompatibility of oxidized-dextran crosslinked hemoglobin (Odex-Hb MPs) as evidenced by their hemolysis ratio of less than 5%, inertness to leukocyte phagocytosis, and lack of platelet activation [11]. Okamoto W et al. developed hemoglobin covalently coated with poly(2-ethyl-2-oxazoline) (POx-Hb) and evaluated its hemocompatibility, demonstrating that it has no adverse effects on platelet activation or the phagocytic function of granulocytes and monocytes [12]. Gizem B et al. reported the hemocompatibility of hemoglobin-loaded PEGylated ZIF-8 nanoparticles. The results indicated that the nanoparticles did not affect activated partial thromboplastin time (APTT) or prothrombin time (PT), did not activate the complement system, and exhibited a hemolysis rate of less than 5% even at 4 mg/mL [13]. However, there have been no reports on the hemocompatibility of surface-modified HBOCs where PEG is directly conjugated to hemoglobin.

Additionally, current hemocompatibility assessments of HBOCs rely predominantly on in vitro models that assess interactions between HBOCs and isolated blood components under static conditions and cannot fully simulate the physiological environment. In vivo, HBOCs must interact simultaneously with multiple blood components under blood flow in the presence of vascular endothelium. Yunfan Pan et al. demonstrated that flow influences the patterns of erythrocyte damage [14]. The vascular endothelium maintains blood homeostasis by secreting substances that regulate coagulation and erythrocyte function. For example, anticoagulant mediators such as prostacyclin (PGI_2_) and nitric oxide (NO) inhibit platelet activation, tissue-type plasminogen activator (t-PA) promotes thrombolysis, and heparan sulfate enhances the activity of antithrombin III. Conversely, procoagulant factors, including von Willebrand factor (vWF), mediate platelet adhesion, and tissue factor (TF) initiates the extrinsic coagulation pathway. Among these, NO plays a central role in maintaining erythrocytes’ deformability, inhibiting aggregation, and indirectly regulating oxygen transport [15,16]. Therefore, static in vitro models fail to recapitulate the complex dynamic processes that occur under blood flow, involving interactions among HBOCs, multiple blood components, and the vascular endothelium.

Moreover, erythrocytes serve as the foundation for ensuring oxygen transport. If erythrocytes are disrupted or their oxygen supply efficiency decreases, it may cause tissue hypoxia, even leading to organ damage (e.g., the kidneys and brain are susceptible to hypoxia) [17]. The effects of HBOCs cooperating with erythrocytes on oxygen transport also play a critical role in hemocompatibility. Therefore, a combination of in vivo and in vitro studies is essential to comprehensively evaluate the hemocompatibility of PEG-bHb, including its erythrocyte oxygen supply efficiency.

Therefore, the present study evaluated the hemocompatibility of PEG-bHb through a combination of in vitro and in vivo assays. In vitro experiments assessed the impact of PEG-bHb on erythrocyte morphology and function, coagulation, complement activation, and leukocyte phagocytosis. In vivo, Wistar rats were administered PEG-bHb, and systemic outcomes were monitored, including erythrocyte function, coagulation, complement activation, hematologic components, and histopathological changes in major organs. By integrating these comprehensive assessments, this work provides crucial evidence regarding the hemocompatibility of PEG-bHb, thereby supporting its further process optimization and potential clinical translation.

## 2. Result

### 2.1. Size, Zeta Potential, COP, and Viscosity of PEG-bHb

**Figure 1:**
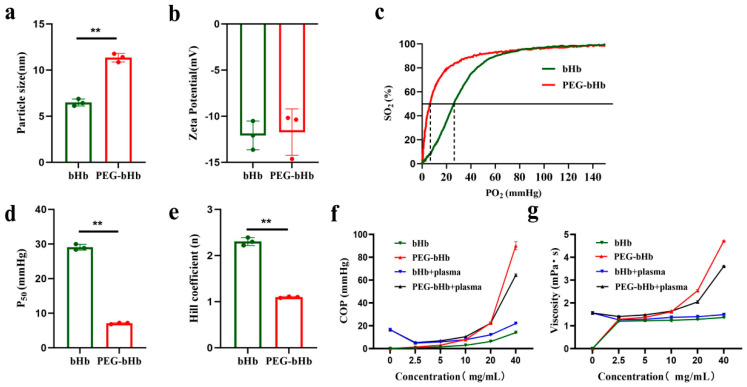
Physicochemical characterizations of bovine hemoglobin (bHb) and polyethylene glycol-conjugated bovine hemoglobin (PEG-bHb). (**a**). Particle size of bHb and PEG-bHb (n = 3). (**b**). Zeta potential of bHb and PEG-bHb. (**c**). Oxygen dissociation curve (ODC) of bHb and PEG-bHb. (**d**). Partial pressure of oxygen at half (P_50_) of bHb and PEG-bHb (n = 3). (**e**). Hill coefficient of bHb and PEG-bHb (n = 3). (**f**). Colloid osmotic pressure (COP) of bHb, PEG-bHb, and their mixtures with plasma (n = 3). (**g**). Viscosity of bHb, PEG-bHb, and their mixtures with plasma (n = 3). (** *p* < 0.01 indicate statistical significance).

As shown in Figure 1a, the hydrated particle size of PEG-bHb was 11.34 ± 0.46 nm, whereas that of unmodified bovine hemoglobin (bHb) was 6.48 ± 0.38 nm. This indicates that the PEG modification led to an approximately 75% increase in the hydrated particle size of bHb. Figure 1b demonstrates that the zeta potential of PEG-bHb was −12.08 ± 1.56 mV, while that of bHb was −11.73 ± 2.52 mV. There was no statistically significant difference between the bHb and PEG-bHb.

The oxygen dissociation curves (ODCs), partial pressure of oxygen at half (P_50_) values, and Hill coefficients for bHb and PEG-bHb are presented in Figure 1c–e. Compared to bHb, PEG-bHb demonstrated a leftward shift in ODC, a significant reduction in both P_50_ and the Hill coefficient.

The COP of bHb, PEG-bHb, bHb+plasma, and PEG-bHb+plasma are illustrated in Figure 1f. The COP of bHb increased slightly with increasing concentration. In contrast, the COP of PEG-bHb increased exponentially with increasing concentration. Notably, when the concentration of PEG-bHb exceeded 20 mg/mL, the increment of its COP became remarkably obvious; the COP of the 40 mg/mL PEG-bHb group was 6.47 times that of bHb. When bHb and PEG-bHb were mixed with plasma, the results demonstrated that PEG-bHb exhibited a potent capacity to increase COP compared to bHb.

The viscosity of bHb, PEG-bHb, bHb+plasma, and PEG-bHb+plasma is presented in Figure 1g. The viscosity of bHb barely changed with increased concentration. In contrast, the viscosity of PEG-bHb increased with concentration and followed a distinct exponential trend. The viscosity of the 40 mg/mL PEG-bHb group reached 3.45 times that of bHb. When bHb and PEG-bHb were mixed with plasma, the results demonstrated that PEG-bHb exhibited a potent capacity to increase viscosity compared to bHb.

### 2.2. Effects of PEG-bHb on Erythrocyte Morphology, Eryptosis, and Oxygen Supply Efficiency In Vitro

**Figure 2:**
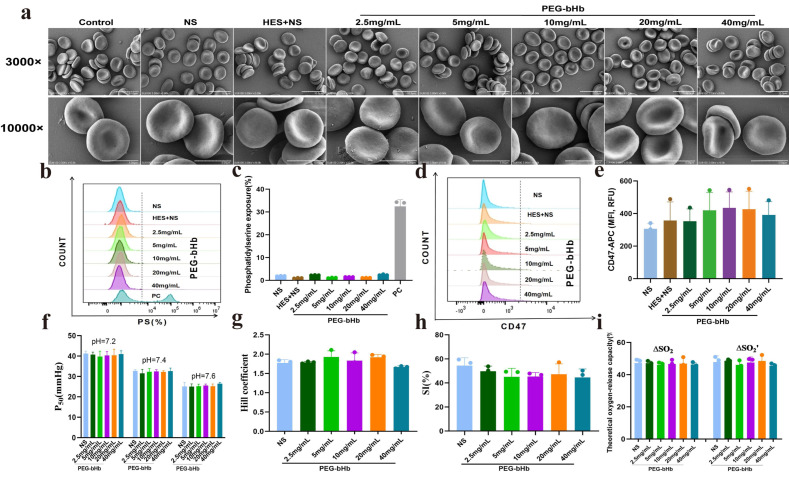
The effect of PEG-bHb on the morphology and function of erythrocyte in vitro. (**a**). Scanning electron microscopy (SEM) of erythrocyte following incubation with PEG-bHb (The scale bar for the 3000× image represents 10 μm, while the scale bar for the 10,000× image represents 5 μm). (**b**). Flow cytometry histogram of phosphatidylserine (PS) exposure of erythrocyte following incubation with PEG-bHb. (**c**). Percentage of PS exposure rate of erythrocyte following incubation with PEG-bHb (n = 3). (**d**). Flow cytometry histogram of CD47 on erythrocyte following incubation with PEG-bHb. (**e**). Fluorescence intensity of CD47 on erythrocyte following incubation with PEG-bHb (n = 3). (**f**). P_50_ of erythrocyte following incubation with PEG-bHb (n = 3). (**g**). Hill coefficient of erythrocyte following incubation with PEG-bHb (n = 3). (**h**). Sensitivity index (SI) of erythrocyte following incubation with PEG-bHb (n = 3). (**i**). Theoretical oxygen release capacity of erythrocyte following incubation with PEG-bHb (n = 3). (Plain environment theoretical oxygen release capacity(∆SO_2_), and plateau environment theoretical oxygen release capacity (∆SO_2_′)).

As shown in Figure 2a, compared with control, erythrocytes incubated with NS (normal saline), HES+NS (crystal: colloid = 3:1; crystal is NS; colloid is Hydroxyethyl starch 130/0.4 sodium chloride injection), and 2.5, 5, 10, 20, 40 mg/mL PEG-bHb all exhibited intact cell membranes, clear boundaries, and normal cell morphology.

The phosphatidylserine (PS) exposure profiles (Figure 2b,c) demonstrated that erythrocytes incubated with HES+NS and 2.5, 5, 10, 20, 40 mg/mL PEG-bHb showed a level of PS exposure comparable to the NS group, and significantly lower than that of the positive control. The CD47 fluorescence intensity of erythrocytes in the groups treated with HES+NS and 2.5, 5, 10, 20, 40 mg/mL PEG-bHb was identical to that of the groups treated with NS, as shown in Figure 2d,e.

Figure 2f–i showed the P_50_, Hill coefficient, sensitivity index (SI), and theoretical oxygen release capacity of erythrocytes incubated with NS, 2.5, 5, 10, 20, and 40 mg/mL PEG-bHb. No statistically significant differences were observed in these parameters across all experimental groups, indicating that PEG-bHb does not affect erythrocyte oxygen supply efficiency.

### 2.3. Effects of PEG-bHb on Coagulation Function In Vitro

**Figure 3:**
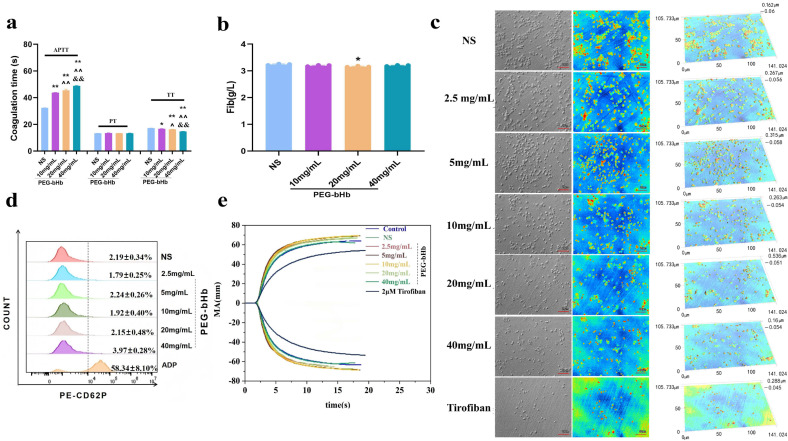
The effect of PEG-bHb on coagulation in vitro. (**a**). Activated partial thromboplastin time (APTT), prothrombin time (PT), and thrombin Time (TT) of platelet-poor plasma (PPP) following incubation with PEG-bHb (n = 3, technical replicates). (**b**). Fibrinogen (Fib) of PPP following incubation with PEG-bHb (n = 3, technical replicates). (**c**). Platelet adhesion of platelet-rich plasma (PRP) following incubation with PEG-bHb. (**d**). Flow cytometry histogram of platelet activation in PRP following incubation with PEG-bHb (n = 3, technical replicates). (**e**). Thromboelastography (TEG) of the whole blood following incubation with PEG-bHb. (* *p* < 0.05, ** *p* < 0.01, compared with NS; ^^^ *p* < 0.05, ^^^^ *p* < 0.01, compared with 10 mg/mL PEG-bHb; ^&&^ *p* < 0.01, compared with 20 mg/mL PEG-bHb).

Plasma coagulation parameters, including APTT, PT, thrombin time (TT), and fibrinogen (Fib), were measured to evaluate the effect of PEG-bHb on plasma coagulation functions. As shown in Figure 3a, 10, 20, and 40 mg/mL PEG-bHb groups significantly prolonged plasma APTT compared to the NS group (all ** *p* < 0.01). The APTT in the 20 and 40 mg/mL PEG-bHb groups was significantly higher than that in the 10 mg/mL PEG-bHb group (both ^^^^ *p* < 0.01), and the APTT in the 40 mg/mL PEG-bHb group was higher than that in the 20 mg/mL PEG-bHb group (^&&^ *p* < 0.01). Compared with the NS group, the TT was significantly shortened in the 10, 20, and 40 mg/mL PEG-bHb groups (* *p* < 0.05, ** *p* < 0.01 and ** *p* < 0.01, respectively). The TT in the 20 mg/mL PEG-bHb group was shorter than that in the 10 mg/mL PEG-bHb group (^^^ *p* < 0.05), and the TT in the 40 mg/mL PEG-bHb group was significantly shorter than those in the 10 mg/mL and 20 mg/mL PEG-bHb groups (^^^^ *p* < 0.01 and ^&&^ *p* < 0.01). In addition, PEG-bHb had no significant effect on PT in all dosage groups. Although a statistically significant reduction in fibrinogen level was observed at 20 mg/mL compared to the NS group (* *p* < 0.05), the absolute change was biologically insignificant, showing a marginal decrease in mean values from 3.24 to 3.16 (Figure 3b).

The effect of PEG-bHb on platelet adhesion is presented in Figure 3c. The results showed that when the PEG-bHb concentration exceeded 20 mg/mL, the number of adherent platelets decreased gradually, and platelet spreading behavior weakened with increasing PEG-bHb concentration. However, it is still better than the tirofiban group.

The effect of PEG-bHb on platelet activation was evaluated by P-selectin (CD62P) positive expression (Figure 3d). The CD62P-positive rates in samples treated with 2.5, 5, 10, 20, and 40 mg/mL PEG-bHb showed no significant difference compared with the NS; meanwhile, the CD62P expression in all PEG-bHb groups was significantly lower than that in the ADP-positive control group.

The effect of PEG-bHb on whole blood coagulation was evaluated by thromboelastography (TEG) (Figure 3e, Table 1). Compared with the normal whole blood control (NC), no significant changes in R, K, Angle, or MA were observed in the NS group or in groups treated with 2.5–40 mg/mL PEG-bHb. At 40 mg/mL PEG-bHb, the EPL increased to 9.5%, which was higher than that in the NC group but remained below the critical threshold of 15% (per the device manufacturer’s specifications). All experimental groups showed no change in the R value, an increase in MA, and decreases in the Angle and K values compared to the tirofiban group.

### 2.4. Effects of PEG-bHb on Complement Component 3a (C3a), Complement Component 5a (C5a), and Leukocyte Phagocytic Function In Vitro

**Figure 4:**
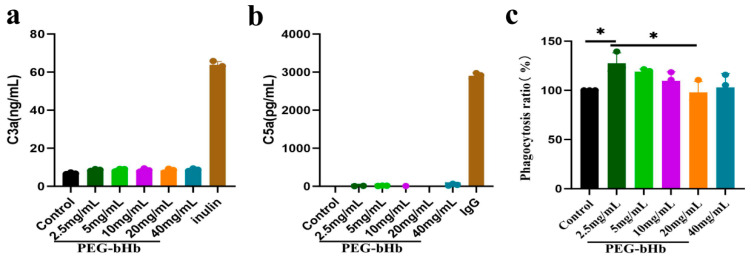
Effects of PEG-bHb on complement and leukocyte phagocytosis in vitro. (**a**). complement component 3a (C3a) of PPP following incubation with PEG-bHb (n = 3, technical replicate). (**b**). complement component 5a (C5a) of PPP following incubation with PEG-bHb (n = 3, technical replicate). (**c**). Leukocyte phagocytosis of Raw 264.7 cells on following incubation with PEG-bHb (n = 3). (* *p* < 0.05, indicates statistical significance.).

The effects of PEG-bHb on complement system activation were evaluated by measuring the concentrations of C3a and C5a (Figure 4a,b). Inulin and immunoglobulin G (IgG) were used as positive controls for C3a and C5a, respectively. The results showed that the concentration of C3a in plasma exposed to 2.5, 5, 10, 20, and 40 mg/mL PEG-bHb did not differ from that in the control group and was significantly lower than that in the inulin group. Similarly, the concentration of C5a in plasma exposed to 2.5, 5, 10, 20, and 40 mg/mL PEG-bHb did not differ from that in the control group and was significantly lower than that in the IgG group. These results indicate that PEG-bHb at 2.5, 5, 10, 20, and 40 mg/mL did not affect complement activation.

The phagocytic function of leukocytes in the presence of PEG-bHb was assessed (Figure 4c). Using the untreated control (set as 100%) as a reference, a significant increase in phagocytosis was observed at 2.5 mg/mL PEG-bHb (* *p* < 0.05). In contrast, no statistically significant differences were detected at 5, 10, 20, and 40 mg/mL PEG-bHb compared with the control. Furthermore, although the phagocytic rate at 20 mg/mL was lower than that at 2.5 mg/mL, no additional dose-dependent reduction was observed at higher concentrations. These results indicate that 2.5 mg/mL PEG-bHb can promote leukocyte phagocytic function, whereas 5, 10, 20, and 40 mg/mL PEG-bHb do not affect leukocyte phagocytic function compared with the control group.

### 2.5. Effects of Intravenous Administration of PEG-bHb on Erythrocyte Oxygen Supply Efficiency and Eryptosis In Vivo

**Figure 5:**
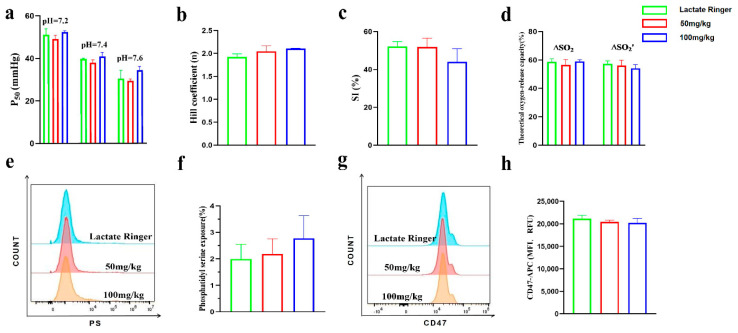
The effect of PEG-bHb on the function of erythrocyte in vivo. (**a**). P_50_ of whole blood (n = 3). (**b**). Hill coefficient of whole blood (n = 3). (**c**). SI of whole blood (n = 3). (**d**). Theoretical oxygen release capacity of whole blood (n = 3). (**e**). Flow cytometry histogram of PS exposure of erythrocyte (n = 4). (**f**). Percentage of PS exposure rate of erythrocyte (n = 4). (**g**). Flow cytometry histogram of CD47 on erythrocyte. (**h**). Fluorescence intensity of CD47 on erythrocyte (n = 4).

The oxygen supply efficiency of whole blood following PEG-bHb administration in vivo are summarized in Figure 5a–d. The P_50_ values (Figure 5a), Hill coefficients (Figure 5b), SI (Figure 5c), and theoretical oxygen release capacities (Figure 5d) in rats treated with 50 and 100 mg/kg PEG-bHb showed no statistically significant differences compared to the lactated Ringer’s control group. These results indicate that intravenous infusion of PEG-bHb, across the tested doses, did not alter the oxygen supply efficiency.

To assess the effect of PEG-bHb on erythrocyte eryptosis, PS exposure and CD47 expression were examined. The flow cytometric histograms for PS showed overlapping profiles across all groups (Figure 5e), and the percentage of PS-positive erythrocytes in the 50 and 100 mg/kg PEG-bHb groups showed no significant difference compared to the lactated Ringer’s control (Figure 5f). Similarly, CD47 expression on erythrocytes remained consistent among groups, as evidenced by flow cytometric histograms (Figure 5g) and comparable fluorescence intensity values (Figure 5h). Collectively, these data indicate that PEG-bHb does not induce PS exposure or alter the surface expression of CD47 on erythrocytes.

### 2.6. Effects of Intravenous Administration of PEG-bHb on Coagulation Function and Complement Activation In Vivo

**Figure 6:**
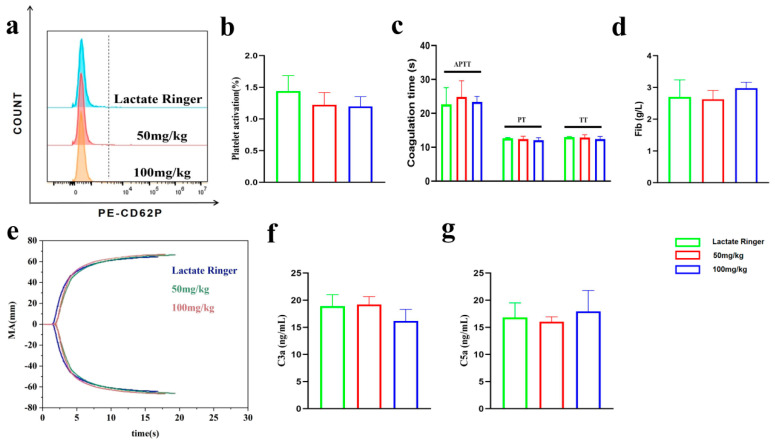
Effects of PEG-bHb on coagulation function and complement activation in vivo. (**a**). Flow cytometric histogram of platelet activation. (**b**). Percentage of platelet activation (n = 4). (**c**). APTT, PT, and TT of PPP (n = 4). (**d**). Fib of PPP (n = 4). (**e**). The TEG of the whole blood. (**f**). The level of C3a (n = 3). (**g**). The level of C5a (n = 3).

As shown in the flow cytometry histogram and quantitative analysis (Figure 6a,b), no statistical differences were observed among the groups, indicating that PEG-bHb administration did not induce platelet activation in vivo.

The APTT, PT, TT and Fib parameters of PPP are presented in Figure 6c,d. No significant differences were observed among the groups, suggesting that the coagulation function of PPP was not significantly altered by in vivo administration of PEG-bHb.

The TEG curves and parameters of whole blood are presented in Figure 6e and Table 2. No significant differences were observed among the groups, suggesting that the coagulation function was not significantly altered by in vivo administration of PEG-bHb.

The plasma concentration of complement component C3a and C5a is summarized in Figure 6f,g. No significant differences were observed among the groups, suggesting that the complement system was not activated by in vivo administration of PEG-bHb.

### 2.7. Effects of Intravenous Administration of PEG-bHb on Blood Composition and Key Organs

The complete blood count and blood gas analysis showed no significant changes following PEG-bHb administration. The data are presented in Figures S1 and S2 (see Supplementary Materials).

**Figure 7:**
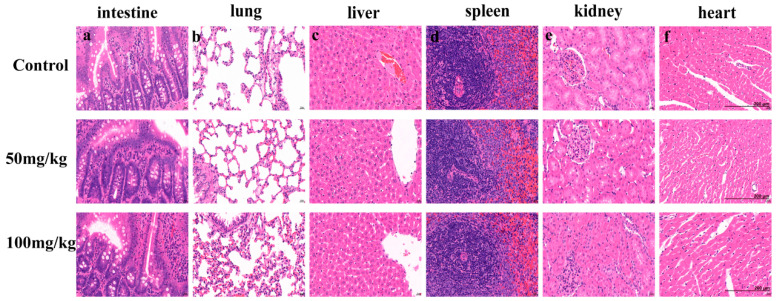
The effect of PEG-bHb on internal organs. Representative hematoxylin and eosin (H&E)-stained tissue sections from rats treated with Lactated Ringer’s solution (Control) or PEG-bHb at doses of 50 mg/kg and 100 mg/kg. (**a**). Intestine. (**b**). Lung. (**c**). Liver. (**d**). Spleen. (**e**). Kidney. (**f**). Heart. Scale bar (applicable to all panels): 200 µm.

Since blood components interact with tissues and organs, investigating the effect of PEG-bHb on key organs is also an important indicator for evaluating the hemocompatibility of PEG-bHb. As shown in Figure 7a–f, administration of PEG-bHb at 50–100 mg/kg produced no observable histopathological alterations in the intestine, lung, liver, spleen, kidney, or heart compared with controls. Specifically, intestinal villi remained intact without detachment or structural abnormalities; lung tissues maintained clear demarcation between interstitium and parenchyma; hepatic architecture including central veins, hepatic cords, sinusoids, perisinusoidal spaces and bile ducts exhibited preserved morphology; splenic structures comprising the capsule, trabeculae, white pulp, red pulp and marginal zone remained intact; renal glomeruli and tubules showed clearly delineated structures; and myocardial tissue displayed normal texture without pathological changes. Importantly, no inflammatory cell infiltration or immune complex deposition was detected in any of the examined organs. Collectively, these results demonstrate that PEG-bHb at doses ≤ 100 mg/kg does not induce pathological necrosis or significant tissue damage in vital organs.

## 3. Discussion

As a kind of intravenous Large Volume Infusion, HBOCs must demonstrate safety with blood components. A comprehensive and systematic evaluation of hemocompatibility is essential to confirm its safety, which, in turn, determines its application in critical clinical scenarios such as trauma and hemorrhagic shock [18].

PEG-bHb exhibits distinct physicochemical properties, including an increased hydrodynamic diameter, negative surface charge, high COP, elevated viscosity, and a reduced P_50_. PEG-bHb exhibits an approximately 75% larger hydrodynamic diameter than native bHb, a characteristic that reduces vasoactivity and avoids rapid renal clearance. The negatively charged surface of PEG-bHb electrostatically repels both blood cells and plasma albumin, thereby avoiding cell damage and the formation of a protein corona. Furthermore, PEG-bHb exhibits approximately the COP and viscosity of MP4 at equivalent concentrations [19]. The elevated COP augments blood volume, while the increased viscosity offsets the hemodilution-induced viscosity reduction during hemorrhagic shock, which makes it particularly beneficial for hemorrhagic shock patients, a condition in which concurrent volume resuscitation and oxygen transport are essential [20]. In terms of oxygen-binding properties, PEG-bHb displays a P_50_ comparable to MP4, exhibiting a left-shifted ODC consistent with heightened oxygen affinity, which promotes efficient oxygen loading in the lungs, while the Bohr effect facilitates oxygen offloading in hypoxic tissues [21]. Given the similarity with MP4, the concentration of PEG-bHb both in vitro and in vivo studies were consistent with that of MP4. According to a rat hemodilution experiment [22], the in vitro concentration range of PEG-bHb was set as 2.5–40 mg/mL. For in vivo experiments, doses of 50–100 mg/kg were selected with reference to phase I clinical data of MP4 [23].

The hemocompatibility of PEG-bHb with erythrocytes, the most abundant blood cells, is fundamental to its safety and efficacy. Scanning electron microscope (SEM) analysis confirmed intact erythrocyte membranes with a normal biconcave discoid morphology, indicating no significant alteration in erythrocyte morphology. Hemolysis and eryptosis represent two distinct pathways of erythrocyte injury or death, both of which are ultimately eliminated by the phagocytic system, including macrophages [24]. Hemolysis leads to the release of free hemoglobin, which not only exerts nephrotoxic effects but also enhances platelet activation [25]. In accordance with ISO 10993-4:2017/Amd 1:2025, which stipulates a hemolysis threshold below 5% for biomaterials. Although the intrinsic color of PEG-bHb hindered the direct assessment of hemolysis, the absence of significant hemolysis was confirmed by both intact erythrocyte morphology and unaltered erythrocyte counts (in Supplementary Figure S2) in rats administered at doses of 50–100 mg/kg. Eryptosis, a programmed cell death mechanism, is crucial for maintaining blood homeostasis. Our in vitro experiments showed that incubation of erythrocytes with 2.5–40 mg/mL PEG-bHb resulted in PS exposure rates and CD47 expression levels comparable to the NS control group. Consistent with these findings, in vivo administration of PEG-bHb (50–100 mg/kg) in rats did not elevate PS exposure and did not alter CD47 expression. The material’s intrinsic surface properties may explain the observed hemocompatibility. Its anionic surface induces electrostatic repulsion against the similarly charged erythrocyte membranes, thereby mitigating erythrocyte damage and subsequent hemolysis, a finding consistent with the hemocompatibility optimization strategy reported by J. A. Roacho-Perez et al. for negatively charged dendrimers and PEGylated nanoparticles [26]. The oxygen supply efficiency of erythrocytes is their most critical function. Yet, the hemocompatibility assessment of previous materials has seldom focused on it, which is characterized by several parameters: P_50_, Hill coefficient, SI, and theoretical oxygen release capacity. This study comprehensively evaluated the oxygen supply efficiency of PEG-bHb using integrated in vitro and in vivo assays. In vitro, after incubation with PEG-bHb, erythrocytes revealed no significant alterations in P_50_, Hill coefficient, SI, and theoretical oxygen release capacity, indicating that PEG-bHb does not perturb the hemoglobin-oxygen-binding of erythrocytes. Consistent with these findings, in vivo evaluation further confirmed that whole-blood oxygen supply efficiency remained unaltered following 50–100 mg/kg PEG-bHb administration. Together, these findings indicate that PEG-bHb is compatible with erythrocytes and does not impair blood oxygen transport. While this study investigated low-dose PEG-bHb infusion in healthy rats, the actual impact of administering high doses of low-P_50_ PEG-bHb on systemic oxygen supply efficiency remains to be validated through further experiments, particularly when considering the compensatory P_50_ adjustment that follows hemorrhagic shock.

Coagulation function is a critical parameter in the hemocompatibility assessment of HBOCs and may be a major obstacle to their clinical translation. Previous studies have indicated that first-generation HBOC products (e.g., HemAssist, PHP-Hemoximer, and Hemolink) were discontinued during clinical trials due to an increased risk of myocardial infarction. The underlying mechanism involves coagulation abnormalities, such as inducing a hypercoagulable state and promoting thrombosis, which can ultimately trigger myocardial infarction when coronary artery occlusion occurs [27]. In hemorrhagic shock, which is a primary target condition for HBOCs therapy, the blood coagulation system undergoes both adaptive and maladaptive changes. At the site of bleeding, activation of the clotting cascade and platelets leads to the formation of a hemostatic plug. In the absence of injury, fibrinolytic activity is enhanced, likely to prevent microvascular thrombosis. However, excessive plasmin activity combined with autoheparinization due to glycocalyx shedding can lead to pathologic hyperfibrinolysis and a resultant diffuse coagulopathy [17]. If HBOCs further impact coagulation, they could worsen patient outcomes. Therefore, a comprehensive evaluation of the effects of HBOCs on coagulation function is essential for their development.

Coagulation is a highly coordinated cascade reaction, comprising the initiation, amplification, and propagation phases. This study comprehensively evaluated the coagulation process through systematic in vitro and in vivo experiments. In vitro results showed prolonged APTT with unchanged PT, indicating that PEG-bHb may impaire the intrinsic but not the extrinsic coagulation pathway. Concurrently, the shortened TT in the presence of unchanged Fib levels suggested an accelerated conversion of Fib to fibrin. Meledeo et al. reported that in vitro hemodilution with HBOC-201 (glutaraldehyde-crosslinked bHb) induced a dose-dependent reduction in Fib concentration, with increasing PT and APTT [28]. In this study, the 40 mg/mL PEG-bHb group showed a moderately prolonged APTT (48.9 s), which remained below the bleeding risk threshold (APTT > 60 s), while PT and Fib were unaffected. A shortened TT (14.6 s) was recorded, still falling within the normal range (14–21 s). In vivo administration of 50–100 mg/kg PEG-bHb did not affect APTT, PT, TT, or Fib. These results indicate that PEG-bHb exhibits a favorable coagulation compatibility profile, characterized by measurable yet clinically insignificant alterations in vitro and no adverse effects in vivo. In vitro and in vivo experimental settings demonstrated no increase in CD62P positivity, confirming unaffected platelet activation function. Previous reports from Kloypan C and Okamoto W indicate that Odex-Hb MPs and POx-Hb reportedly exert no effect on platelet activation [11,12]. However, no studies have yet investigated the impact of HBOCs on platelet adhesion and aggregation. Our in vitro assays revealed that high concentrations of PEG-bHb reduced platelet adhesion, indicating that while platelets retain the capacity to serve as catalytic platforms for coagulation, their adhesive function was impaired. Consequently, it is not feasible to draw a direct link between this particular result and HBOC-related bleeding episodes. TEG results from in vitro and in vivo studies showed no significant alterations in R time, K time, α-angle, or MA value. Although the EPL was mildly elevated to 9.5% at the highest in vitro concentration (40 mg/mL), it remained below the clinically significant threshold of 15%, indicating no substantial compromise in overall clot stability and an absence of hyperfibrinolysis [29]. Alexander P. et al., in their study utilizing an in vitro model, demonstrated that PolyHeme (glutaraldehyde-crosslinked HbA) enhanced fibrinolytic activity in a dose-dependent manner, independent of plasmin. Regardless of the addition of exogenous plasminogen activators (tPA), PolyHeme consistently promoted fibrinolysis, leading to hyperfibrinolysis [30]. It was hypothesized that this phenomenon might be attributable to the structural similarity of PolyHeme to cell-free hemoglobin, which consequently induces coagulopathy and disrupts the haemostatic balance between coagulation and fibrinolysis [31]. Mingzi Lu et al. reported that TEG analysis of hemoglobin-loaded nanoparticles (HbPs) synthesized with mPEG-PLGA as the carrier showed no induction of hyperfibrinolysis, which may be attributed to mPEG-PLGA’s surface shielding effect on hemoglobin. Therefore, the favorable coagulation performance of PEG-bHb can be explained by the surface shielding effect of PEG on hemoglobin. Evaluation by TEG showed that mild APTT prolongation and a slight decrease in platelet adhesion did not disrupt the overall coagulation. These results confirm that PEG-bHb shows no signs of association with thrombotic or hemorrhagic risks, indicating that it is an HBOC with favorable hemocompatibility.

As an exogenous modification material widely used in intravenously administered drugs, the immunogenicity of PEG is a critical factor influencing the clinical translation of biotherapeutics. Immune responses triggered by PEG can not only accelerate drug clearance and shorten half-life but also, more severely, activate the complement system and induce complement activation-related pseudoallergy (CARPA). The post-administration levels of C3a and C5a directly and specifically reflect whether PEG has induced complement activation, thereby providing essential experimental evidence for evaluating the ability of a product to avoid acute immunotoxicities such as CARPA. Therefore, the levels of C3a and C5a are crucial to assess the complement activation potential of PEG-bHb [32]. As a xenobiotic substance, HBOCs may have the risk of activating the complement system or altering leukocyte function, potentially triggering inflammatory or allergic responses [33]. This study demonstrated that 2.5–40 mg/mL PEG-bHb did not significantly affect the levels of complement components C3a and C5a. The concentrations of C3a and C5a were comparable to those in the control group and were substantially lower than those in the PC group (inulin for C3a and IgG for C5a). Furthermore, PEG-bHb administered at 50–100 mg/kg did not increase the concentrations of C3a or C5a in vivo. Critically, preventing complement activation averts vasodilation, increased vascular permeability, and leukocyte chemotaxis, which could significantly exacerbate tissue injury, particularly in vulnerable states such as hemorrhagic shock or trauma. Moreover, the absence of inflammatory cell infiltration in vital organs (intestine, lung, liver, spleen, kidney, and heart) provides additional evidence that PEG-bHb does not provoke systemic inflammation, consistent with the lack of complement activation observed in this study.

The evaluation of leukocyte phagocytic function substantiates the hemocompatibility of PEG-bHb. A mild enhancement of phagocytosis was observed at 2.5 mg/mL PEG-bHb, whereas 5–40 mg/mL PEG-bHb did not alter this function, demonstrating that leukocytes retain their pathogen-clearance capacity at therapeutic concentrations. Notably, this finding holds clinical importance since impaired phagocytosis could elevate infection risk in critically ill patients, who are the target population of HBOCs and are particularly vulnerable to severe outcomes from secondary infections.

### Limitations

The present work primarily focused on erythrocytes, platelets, and leukocytes in hemocompatibility evaluation. In contrast, plasma aspects were confined to coagulation parameters, including APTT, PT, TT, and Fib. Notably, the adsorption of PEG-bHb to plasma proteins, such as albumin and fibrinogen, was not examined due to technical limitations, as the inherent spectral characteristics of hemoglobin interfere with conventional detection methods, including fluorescence and circular dichroism spectroscopy. Future studies will employ techniques such as SDS-PAGE or HPLC to separate and directly analyze the interactions. Alternatively, the adsorption dynamics may be indirectly assessed by quantifying changes in the concentrations of key plasma proteins, such as albumin and fibrinogen.

In addition, although the detection of erythrocyte PS, CD47 and SEM provided a preliminary assessment of erythrocyte membrane integrity, further detections, such as the fluidity of the lipid bilayer, the supportive role of membrane skeletal proteins and the osmotic intracellular-extracellular balance, would be achieved to verify the cellular condition in subsequent experiments.

## 4. Material and Methods

### 4.1. Synthesis and Characterization of PEG-bHb

Bovine hemoglobin (bHb) was purified from erythrocytes as previously described [34]. PEG-bHb was synthesized in our laboratory [35].

Particle size and zeta potential were determined using the nanoparticle size and zeta potential analyzer (Malvern Panalytical, ZETASIZER ADVANCE, UK). Oxygen dissociation curves (ODCs) were plotted using an oxygen-binding-dissociation analyzer (Softron, Bloodox-2018, Beijing, China). The COP and viscosity of 0, 2.5, 5, 10, 20, and 40 mg/mL PEG-bHb and mixtures of PEG-bHb (at the same concentrations) with plasma were evaluated using the colloid osmometer (High Energy Teck, OSMOMAT 050, Berlin, Germany) and the rotational viscometer (Anton Paar GmbH, ViscoQC 300, Graz, Austria).

### 4.2. Preparation of Animal and Blood Components

Venous whole blood was collected from a 38-year-old female volunteer, and anti-coagulated with 3.2% sodium citrate. The volunteer signed a written informed consent form. Whole blood was centrifuged (Thermo Scientific, FRESCO 21, Waltham, USA) at 300× *g* for 10 min, and the supernatant was collected as platelet-rich plasma (PRP). The remaining fraction was subsequently centrifuged at 1000× *g* for 10 min, and the resulting supernatant was collected as platelet-poor plasma (PPP). The lower layer was collected as the erythrocyte pellet. For experiments involving human blood components, n = 3 represents technical replicates.

Male Wistar rats (250–280 g) were purchased from Vitalriver (Beijing, China). All experimental procedures were approved by the Laboratory Animal Center of the Academy of Military Medical Sciences ( protocol code IACUC-DWZX-2025-557 and date of approval 7 April 2025). Heparin-anticoagulated arterial blood was collected via carotid artery and was centrifuged at 1000× *g* for 10 min (Thermo Scientific, FRESCO 21, Waltham, USA). After discarding the plasma, the erythrocytes were washed with normal saline (NS; No.4 Pharmaceutical, Shijiazhuang, China) and adjusted to a 40% hematocrit (Hct) suspension.

### 4.3. Incubation with PEG-bHb In Vitro

For erythrocyte morphology, eryptosis and oxygen supply efficiency assays, the erythrocyte suspension was mixed with 2.5, 5, 10, 20, and 40 mg/mL PEG-bHb, NS, HES+NS (crystal: colloid = 3:1; crystal is NS; colloid is Hydroxyethyl starch 130/0.4 sodium chloride injection (Fresenius Kabi, Runcorn, UK)), using a 3:7 volume ratio, followed by static incubation at 37 °C for 30 min. The eryptosis induction kit (Beyotime, Shanghai, China) was used as the positive control for eryptosis assays.

For platelet activation and adhesion assays, PRP was incubated with 2.5, 5, 10, 20, and 40 mg/mL PEG-bHb at a 1:2 ratio and shaken at 37 °C for 1 h. Adenosine diphosphate (ADP; TargetMol, Wellesley Hills, USA) and tirofiban (TargetMol, Wellesley Hills, USA) served as positive controls, respectively.

For coagulation parameters (APTT, PT, TT, Fib) assays, PPP was mixed with 10, 20, and 40 mg/mL PEG-bHb in a 9:1 ratio and incubated with shaking at 37 °C for 1 h.

For thromboelastography (TEG) assays, whole blood samples were mixed with 2.5, 5, 10, 20, and 40 mg/mL PEG-bHb at a 9:1 ratio and incubated with shaking at 37 °C for 1 h. The positive control was tirofiban.

For complement activation (C3a, C5a) assays, PPP was mixed with 2.5, 5, 10, 20, and 40 mg/mL PEG-bHb in a 1:2 ratio and incubated with shaking at 37 °C for 1 h. Inulin (MCE, Monmouth Junction, USA) and human IgG (Solarbio, Beijing, China) served as positive controls for C3a and C5a assays, respectively.

For phagocytosis function assays, Raw 264.7 cells were incubated with PEG-bHb samples at 2.5, 5, 10, 20, and 40 mg/mL at 5% CO_2_ and 37 °C for 4 h.

### 4.4. Sample Collection and Measured Parameters In Vivo

Male Wistar rats (250–280 g, n = 12) were randomly divided into three groups (n = 4 per group). The control group received lactated Ringer’s solution (Qitaidb, Qiqihaer, China) intravenously at a volume of 2.5 mL/kg. The treatment groups received intravenous infusions of PEG-bHb at doses of 50 or 100 mg/kg, at a volume of 2.5 mL/kg. Blood samples were collected from Wistar rats 2 h after infusion and were carried out for the analysis of phosphatidylserine (PS) exposure, CD47, platelet activation. At 24 h post-infusion, whole blood was drawn into heparinized tubes for analysis with the automatic hematology analyzer (Mindray, BC-5000VET, Shenzhen, China) and the blood gas analyzer (Radiometer, ABL90 FIEX, Copenhagen, Denmark), which were also analyzed for APTT, PT, thrombin time (TT), and Fibrinogen (Fib).

Male Wistar rats (250–280 g, n = 9) were randomly divided into three groups (n = 3 per group). The infusion, blood sample collection, complete blood count and blood gas analysis procedures were identical to those described above. Blood samples taken 2 h after infusion were analyzed for ODCs, TEG parameters, C3a and C5a. At 24 h post-infusion, the intestines, lungs, livers, spleens, kidneys, and hearts of rats were collected, fixed in 4% paraformaldehyde for 24 h, and then subjected to pathological examination.

### 4.5. Erythrocyte Morphology Observation

Erythrocytes after incubation with PEG-bHb were used for the scanning electron microscope (SEM) assay in vitro. The erythrocyte pellet was suspended in 2.5% glutaraldehyde fixative (Labgic, Beijing, China), and subsequent detection was performed by SEM (Hitachi, SU8100, Tokyo, Japan).

### 4.6. Eryptosis Assay

Erythrocytes after incubation and those from rats infused with PEG-bHb were used for PS and CD47 assays in vitro and in vivo, respectively. About 50,000–100,000 erythrocytes were resuspended in 195 μL of Annexin V-FITC binding buffer, followed by the addition of 5 μL Annexin V-FITC (Beyotime, Shanghai, China). Another 50,000–100,000 erythrocytes were resuspended in 195 μL PBS, with 5 μL CD47 antibody-APC (AAT Bioquest, Pleasanton, USA) added subsequently. Both reaction mixtures were incubated at 25 °C for 20 min in the dark. After incubation, all samples were analyzed using the flow cytometer (Beckman Coulter, CytoFLEX, Brea USA).

### 4.7. Erythrocyte Oxygen Supply Efficiency

Erythrocytes after incubation and the whole blood from rats infused with PEG-bHb were used for ODC assays in vitro and in vivo, respectively. ODCs were plotted at pH 7.2, pH 7.4, and pH 7.6. From ODC, the P_50_ value (Partial pressure of oxygen at half) and the Hill coefficient were obtained, and the acid-base sensitivity index (SI), plain environment theoretical oxygen release capacity (∆SO_2_), and plateau environment theoretical oxygen release capacity (∆SO_2_′) were calculated according to Equations (1)–(3) [36].

Acid-base sensitivity index (SI):

SI= (P_50 (pH 7.2)_ − P_50 (pH 7.6)_)/P_50 (pH 7.4)_ ×100% (1)

Plain environment theoretical oxygen release capacity (∆SO_2_):

ΔSO_2_ = SO_2_ (pH = 7.6, PO_2_ = 100 mmHg) − SO_2_ (pH = 7.2, PO_2_ = 40 mmHg) (2)

Plateau environment theoretical oxygen release capacity (∆SO_2_′):

ΔSO_2_′ = SO_2_ (pH = 7.6, PO_2_ = 60 mmHg) − SO_2_ (pH = 7.2, PO_2_ = 30 mmHg) (3)

### 4.8. Platelet Activation and Adhesion Assay

PRP after incubation and that from rats infused with PEG-bHb were used for platelet activation assays in vitro and in vivo, respectively. Platelet activation was assessed by measuring the expression of P-selectin (CD62p). 5 μL of PRP was added to 25 μL of PBS (containing 1% BSA), followed by 5 μL of PE-CD62P antibody (Biolegend, San Diego, USA). The mixture was incubated in the dark at 25 °C for 15 min, then fixed with 0.5 mL of pre-cooled 1% paraformaldehyde (Seville, Wuhan, China) at room temperature for 10 min, and the percentage of CD62P-positive platelets was determined by flow cytometry.

PRP after incubation with PEG-bHb was used for platelet adhesion assays in vitro. Platelet adhesion was evaluated on glass slides at 37 °C for 1 h, followed by removal of non-adherent platelets through NS washing, fixation with 4% paraformaldehyde, drying, and examination under the laser 3D microscope (Keyence, VK-150K, Osaka, Japan) at 2000× magnification.

### 4.9. APTT, PT, TT, and Fib Assay

PPP after incubation and that from rats infused with PEG-bHb were used for APTT, PT, TT, and Fib assays in vitro and in vivo. APTT, PT, TT, and Fib were analyzed using an automatic coagulation analyzer (Stago, STA-R Max, Asnières-sur-Seine, France) with matching STA^®^ coagulation method kits in vitro and using an animal-use automatic biochemical analyzer (MNCHIP, CelercareV5, Tianjin, China) with matching coagulation four-item assay cartridge in vivo.

### 4.10. TEG Assay

Whole blood after incubation and that from rats infused with PEG-bHb were used for TEG assays in vitro and in vivo, respectively. The TEG assay was performed using a TEG analyzer (Haemonetics, HAS-300, Boston, USA). After detection, TEG curves were plotted, and clotting time (R), clot formation time (K), clot formation rate (Angle), maximum amplitude (MA), estimated percentage of lysis (EPL), and 30-min lysis (LY30) parameters were recorded.

### 4.11. Complement Activation Assay

PPP after incubation, and that from rats infused with PEG-bHb were used for complement activation assays in vitro and in vivo, respectively. The concentrations of complement activation products (C3a and C5a) were detected using the C3a and C5a ELISA Kits (Byabscience, Nanjing, China).

### 4.12. Phagocytosis Function Assay

Raw 264.7 cells after the incubation were used for phagocytic function assays in vitro. The phagocytic function of Raw 264.7 cells was detected using a phagocytosis detection kit (Thermo Fisher Scientific, Waltham, USA).

### 4.13. Statistical Analysis

Flow cytometry data were analyzed using FlowJo software. Cellular debris was excluded by gating on SSC-A versus FSC-A, and doublets/aggregates were removed using FSC-H versus FSC-A and SSC-H versus SSC-A. In the positive control, the left population represented negative cells, while the right population represented positive cells. The positive rate was determined via the fluorescence threshold defined by the negative control population or the minimum point between histogram peaks.

All data are presented as mean ± standard deviation (SD). GraphPad Prism 10.1.2 software was used for graphing and statistical analysis. Differences among groups were analyzed using a one-way ANOVA, followed by an appropriate post hoc test if the ANOVA was significant. If the data violated the assumptions of normality or homogeneity of variance, the non-parametric Kruskal-Wallis test was used instead. Statistical significance was defined as * *p* < 0.05 and ** *p* < 0.01.

## 5. Conclusions

PEG-bHb effectively maintained erythrocyte integrity without inducing eryptosis, while oxygen supply efficiency was unchanged in vitro and in vivo. PEG-bHb exhibited slight effects on systemic coagulation. Additionally, it did not activate complement, induced only transient phagocytic enhancement at 2.5 mg/mL, and did not cause pathological infiltration in vital organs. Overall, these results demonstrate excellent hemocompatibility and support the translational potential of PEG-bHb for clinical use in blood shortages or transfusion-restricted scenarios.

## Figures and Tables

**Table 1:** Clotting kinetics values of whole blood mixed with PEG-bHb.

	R (s)	K (s)	Angle (°)	MA (mm)	A (mm)	EPL (%)	LY30 (%)
NC	3	1.3	74	70	70.3	0	0
NS	2.9	1	78.5	74.9	75.3	0	0
2.5 mg/mL	3	0.9	78	72.3	73	0	0
5 mg/mL	2.9	1	78.1	75.4	75.8	0	0
10 mg/mL	3	1	77.6	75.6	76	0	0
20 mg/mL	3	1	76	73	73.5	0	0
40 mg/mL	2.9	1.2	74.2	68.6	68	9.5	0
2 µM tirofiban	2.8	1.9	68.1	58.7	59.3	0	0

**Table 2:** Effect of PEG-bHb administration on whole blood clotting kinetics in vivo.

	R (s)	K (s)	Angle (°)	MA (mm)	A (mm)	EPL (%)	LY30 (%)
Lactate Ringer	2.6 ± 0.3	0.9 ± 0.1	78.6 ± 1.7	74.6 ± 2.6	75.2 ± 2.5	0	0
50 mg/kg	2.7 ± 0.7	1.0 ± 0.1	77.1 ± 0.9	73.6 ± 2.8	74 ± 2.6	0	0
100 mg/kg	2.8 ± 0.3	1.0 ± 0.3	77.7 ± 3.2	76.2 ± 1.7	76.6 ± 1.6	0	0

## Data Availability

The data presented in this study are available on request from the corresponding author, due to institutional regulations that restrict public sharing of the dataset.

## Supplementary Materials

The following supporting information can be downloaded at: https://www.mdpi.com/article/10.3390/ijms27031262/s1.
