# Supplementary material for: Hemocompatibility Evaluation of PEGylated Bovine Hemoglobin
Source: Int J Mol Sci. 2026 Jan 27;27(3):1262. doi: 10.3390/ijms27031262 (PMC12898566; doi:10.3390/ijms27031262)
Supplement: Supplementary file 1 [file ijms-27-01262-s001.zip › ijms-4064217-supplementary.pdf]

Hemocompatibility Evaluation of PEGylated Bovine Hemoglobin

Supplementary figure

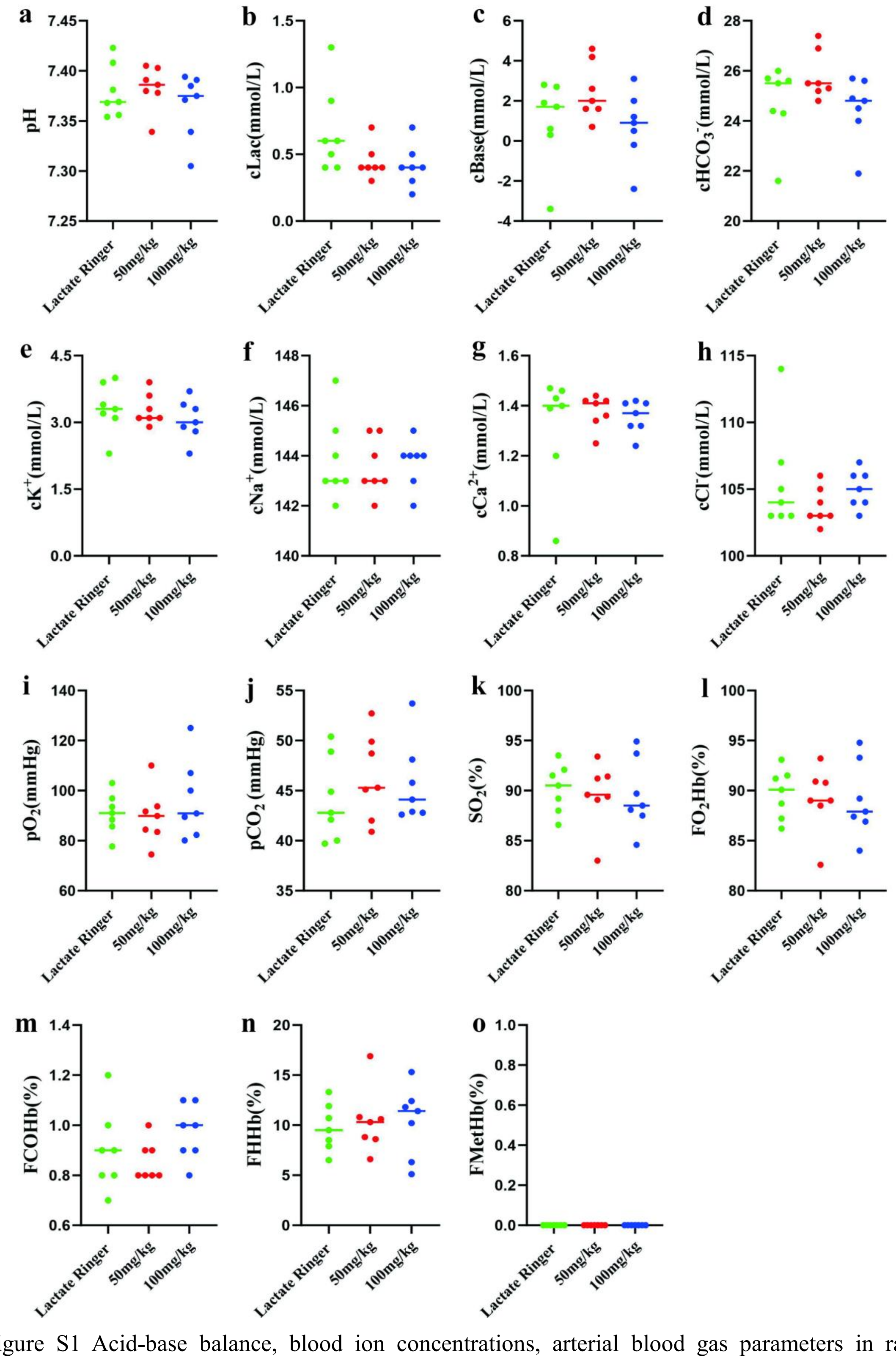

Figure S1 Acid-base balance, blood ion concentrations, arterial blood gas parameters in rats following intravenous infusion of Lactated Ringer's solution and 50–100 mg/kg PEG-bHb. a. pH. b. Lac(mmol/L). c.Base(mmol/L). d. HCO_3^-(mmol/L). e. K^+(mmol/L). f. Na^+(mmol/L). g.

Ca^{2+}(mmol/L). h. Cl^-(mmol/L). i. PO_2(mmHg). j. PCO_2(mmHg). k. SO_2(%.) l. FO_2Hb(%). m. FCOHb(%). n. FHHb(%). o. FMeHb(%).

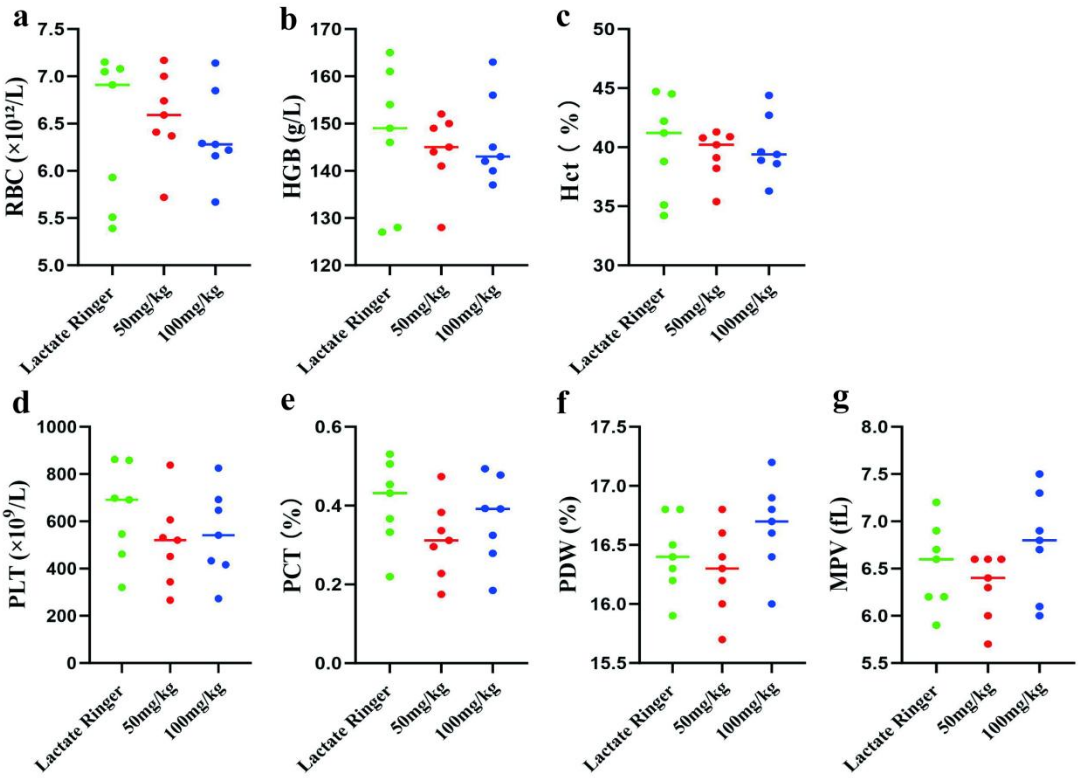

Figure S2 Complete blood count in rats following intravenous infusion of Lactated Ringer's solution and 50–100 mg/kg PEG-bHb. a. RBC($\times 10^{12}$/L). b. HGB(g/L). c. Hct(%). d. PLT($\times 10^9$/L). e. PCT(%). f. PDW(%). g. MPV(fL).
